# Restoring vestibular function during natural self-motion: Progress and challenges

**DOI:** 10.7554/eLife.99516

**Published:** 2024-12-17

**Authors:** Kantapon Pum Wiboonsaksakul, Olivia ME Leavitt Brown, Kathleen E Cullen

**Affiliations:** 1 Department of Biomedical Engineering, Johns Hopkins University School of Medicine Baltimore United States; 2 Kavli Neuroscience Discovery Institute, Johns Hopkins University Baltimore United States; 3 Department of Otolaryngology-Head and Neck Surgery, Johns Hopkins University School of Medicine Baltimore United States; 4 Department of Neuroscience, Johns Hopkins University School of Medicine Baltimore United States; University of Oxford United Kingdom; University of Oxford United Kingdom

**Keywords:** vestibular prostheses, sensory integration, neural plasticity, biomimetic stimulation, neurophysiology

## Abstract

The vestibular system is integral to behavior; the loss of peripheral vestibular function leads to disabling consequences, such as blurred vision, dizziness, and unstable posture, severely limiting activities of daily living. Fortunately, the vestibular system’s well-defined peripheral structure and well-understood encoding strategies offer unique opportunities for developing sensory prostheses to restore vestibular function. While these devices show promising results in both animal models and implanted patients, substantial room for improvement remains. Research from an engineering perspective has largely focused on optimizing stimulation protocol to improve outcomes. However, this approach has often been pursued in isolation from research in neuroscience that has enriched our understanding of neural responses at the synaptic, cellular, and circuit levels. Accordingly, this review bridges the domains of neuroscience and engineering to consider recent progress and challenges in vestibular prosthesis development. We advocate for interdisciplinary approaches that leverage studies of neural circuits at the population level, especially in light of recent advancement in large-scale recording technology, to identify impediments still to overcome and to develop more naturalistic stimulation strategies. Fully integrating neuroscience and engineering in the context of prosthesis development will help advance the field forward and ultimately improve patient outcomes.

## Introduction

**Figure 1.**
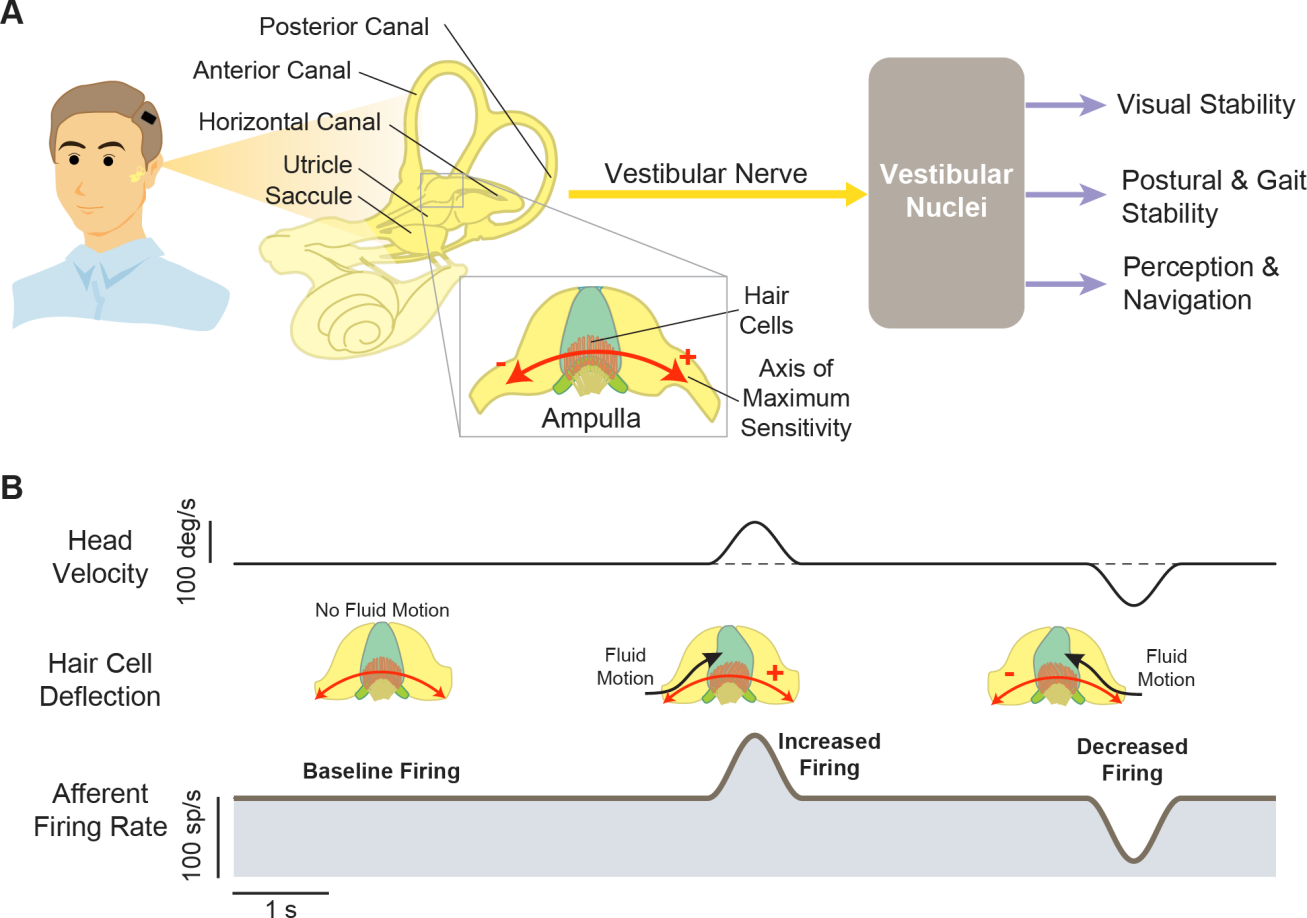
Structures and functions of the vestibular system. (**A**) Three orthogonal semicircular canals (anterior, posterior, and horizontal) provide angular head motion signals, and two otolith organs (utricle and saccule) provide linear acceleration signals. These signals travel via afferent fibers to the vestibular nuclei, where they contribute to pathways involved in visual stability, postural stability, and perception and navigation. Inset: Within the ampulla of each semicircular canal, the hair cells are arranged such that their axis of maximum sensitivity is aligned with the fluid motion direction. (**B**) At rest, afferents fire at a baseline rate (left). During head motion, this baseline is then modulated up (middle) or down (right) to encode head motion in two opposing directions.

The vestibular system detects head motion in space via five peripheral sensory organs located bilaterally in the inner ears: the three semicircular canals, which sense angular rotation in three orthogonal axes, and the two otolith organs, which detect linear acceleration (translational acceleration and gravity) in both horizontal and vertical planes (Figure 1A, left). Together, these peripheral sensors detect six-degree-of-freedom (i.e. three rotational axes and three translational axes) head movement and convey this information to the brain via the VIII nerve. In turn, the brain uses vestibular information to ensure visual and postural stability, as well as an accurate and stable perception of our self-motion and orientation as we move through space (Figure 1A, right). When the vestibular sensors are damaged, these essential functions are disrupted causing disabling symptoms—e.g., blurred vision, dizziness, unstable posture, and gait—which can reduce a patient’s ability to participate in even the simplest of daily activities. It is estimated that more than 1.8 million adults worldwide suffer from bilateral loss of vestibular functions (Boutros et al., 2019a; Schoo et al., 2024). While plasticity does occur within the vestibular pathways following sensory loss (Sadeghi et al., 2012) and can partially improve function (Nashner et al., 1982; Sprenger et al., 2017; Whitney et al., 2016), there had been no effective restorative treatment for those who could not compensate centrally (Sun et al., 2014). An emerging and exciting approach to treat patients is the development of vestibular prostheses. These devices detect head movements and convert them into VIII nerve stimulation, substituting for the damaged peripheral sensors.

The vestibular system’s distinct features make it exceptionally suitable for advancing neuroprosthesis development, with the opportunity to leverage our fundamental understanding of neuroscience to innovate new technologies for individuals with vestibular loss. First, its peripheral structure allows for targeted stimulation encoding each axis of rotation. Second, the encoding strategies of the vestibular nerves are well studied, facilitating the development of biomimetic stimulation. Finally, vestibular reflexes evoke robust compensatory eye and head movements to ensure gaze and postural stability, which are readily measurable as a direct quantification for functional outcomes. Nevertheless, despite our evolving understanding of the vestibular circuits and mechanisms mediating compensation following sensory loss, many recent prosthesis development efforts have focused on surgical implantation techniques or systematically optimizing stimulation waveforms (reviewed in Soto et al., 2023; Stultiens et al., 2023). Importantly, these studies did not consider the fundamental issue of how the brain actually processes and adapts to the information delivered by neural interface stimulation at the levels of neuronal circuits, individual neurons, and synapses.

Indeed, after decades of research and development since their original conception and pioneering animal prototypes in early 2000s (e.g. Gong and Merfeld, 2000; Gong and Merfeld, 2002, see also Wall et al., 2003, for a review), vestibular prostheses remain far from optimal in their ability to restore function, with varying levels of improvement in patients. At this time, there are three ongoing clinical trials focused on evaluating the potential of vestibular implants as a restorative treatment option for patients with such vestibular loss (i.e. University of Geneva and Maastricht University Hospital group [Crétallaz et al., 2020; Perez Fornos et al., 2014; Guinand et al., 2016; Nguyen et al., 2016; Starkov et al., 2020], Johns Hopkins University group [Boutros et al., 2019a; Chow et al., 2021], and the University of Washington group [Phillips et al., 2013; Phillips et al., 2015; Rubinstein et al., 2020]). While these implants help relieve dizziness, reduce visual and postural instability, and improve quality of life in patients with vestibular sensory loss, it is important to emphasize that these essential vestibular functions remain only partially restored (see for example Chow et al., 2021).

In this review, we will bring together the largely separate literatures focused on vestibular neurophysiology, on engineering approaches to optimize stimulation protocols, and on emerging results and challenges of ongoing clinical trials. We will first describe basic physiological principles of vestibular pathways and their implementation in vestibular prostheses. We will next discuss current functional outcomes and limitations in animal research and clinical trials. Finally, we will consider challenges within the field and future directions in light of recent experimental studies, which have provided surprising new insights into how the brain interprets signals from these implants.

## The physiology of vestibular pathways: implications for prosthesis development

To understand how current vestibular prostheses work and how their efficacy can ultimately be improved, it is essential to first consider the physiology of the vestibular periphery and the central pathways that detect, process, and utilize vestibular signals to ensure stable gaze and the maintenance of posture during our daily activities. Accordingly, we begin this review by discussing basic vestibular physiological principles and their implementation (or, more often, lack thereof) in prostheses to restore vestibular functions.

### Peripheral sensors and afferents

As noted above, the vestibular system detects head motion with five peripheral sensory organs: three semicircular canals that sense head rotation and two otolith organs that sense linear acceleration (Figure 1A, left). These sensory organs transmit head motion signals—via vestibular afferents within the VIII nerve—to central neurons within the vestibular nuclei to achieve essential functions (Figure 1A, right). When the sensory cells in the periphery (i.e. hair cells) are damaged, vestibular transduction is impaired, resulting in loss of vestibular functions. The most common known cause of hair cells damage is toxicity from systemic aminoglycoside drugs (e.g. gentamicin; Ward et al., 2013). In addition, those with congenital deafness due to loss of auditory hair cells can also lack properly functioning vestibular hair cells (Delmaghani and El-Amraoui, 2022). Vestibular prostheses aim to bypass these damaged sensors by directly detecting head motions and converting them into electrical stimulation of vestibular afferents. However, while these devices attempt to restore normal vestibular afferent activities during head motions, their development has only been guided to varying degrees by basic vestibular physiological principles listed below.

#### i. The semicircular canal sensors are arranged orthogonally to each other to detect rotational head motion in three dimensions

**Figure 2.**
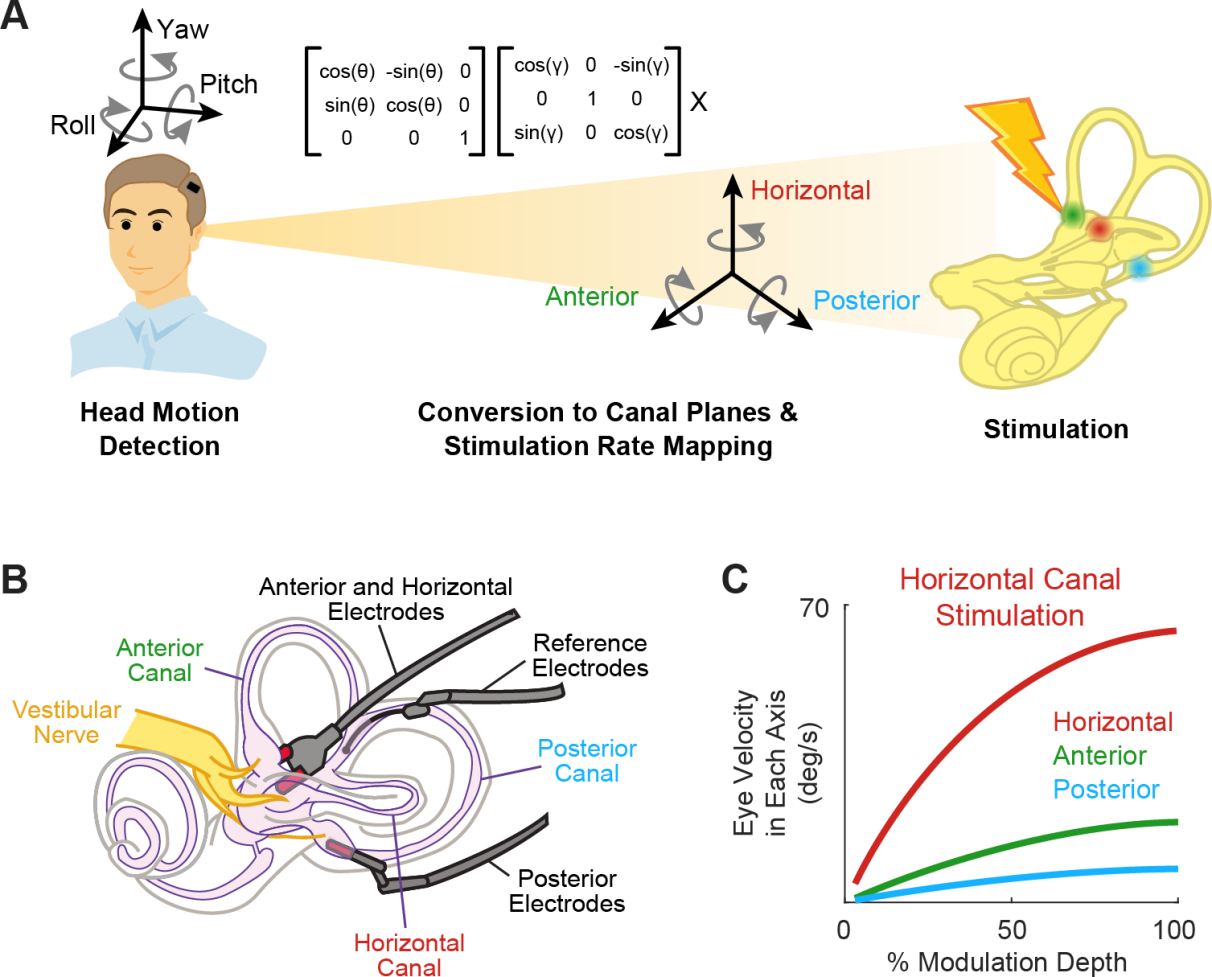
Schematics of the basic functioning of vestibular prostheses. (**A**) To restore rotational vestibular inputs to the brain, vestibular prostheses detect head rotations in three dimensions, transform them onto each canal axis, and map each movement into pulsatile electrical stimulation of each associated branch of vestibular nerve. (**B**) The nerve bundle of each semicircular canals is targeted by an electrode array, allowing for canal-specific stimulation. Adapted from Figure 1 from Chow et al., 2021. (**C**) Eye movement velocity (in each canal axis) evoked by stimulation of the horizontal canal electrode. Adapted from Figure 6 from Boutros et al., 2019a.

To date, nearly all vestibular prostheses have been designed to restore semicircular canal rather than otolith function. This is because the geometric organization and corresponding physiology of semicircular canals makes them particularly well suited for prosthesis development. First, the organization of the three semicircular canals is orthogonal, an arrangement that allows for comprehensive detection of angular head rotation along three distinct axes (reviewed in Cullen, 2019; Figure 1A, left). Additionally, the hair cells within each semicircular canal are aligned such that their polarity corresponds to the primary axis of maximum sensitivity, ensuring optimal detection of angular head movements along each axis (reviewed in Goldberg et al., 2012; Figure 1A and B, red arrows). Moreover, the hair cells in each canal are innervated by a distinct nerve bundle. Taken together, this organization thus allows for axis-specific electrical stimulation via targeted electrode placement within each of the three semicircular canals. Accordingly, it is possible to transform three-dimensional (3D) head motion into a canal-based reference frame by measuring head motion using a 3D gyroscope and then projecting this motion into the three rotational axes of the canals (Figure 2A; Boutros et al., 2019a). Once this is done, head motion in each canal plane can be mapped into a modulated pulse train that is delivered independently to its respective canal electrode to directly activate the associated nerve terminal (Figure 2B). This strategy provides a method for the restoration of semicircular canal function in three dimensions.

Indeed, results from both animal studies and clinical trials have demonstrated the ability to induce 3D reflexive eye movements aligned with the respective canal plane using this approach (Figure 2C; Boutros et al., 2019a; Dai et al., 2011). Due to current spread between electrodes, these responses initially show some misalignment. Fortunately, these small misalignments generally display significant improvement within the first week of continuous stimulation due to adaptation within central vestibular pathways (Dai et al., 2011). Moreover, the brain’s remarkable adaptability to misaligned prosthetic input was directly exemplified in a study by Lewis et al., 2003, where electrical stimulation of one canal plane was deliberately translated into stimulation of a different canal afferent. Despite this discrepancy, the brain adapted to the misaligned stimulation, resulting in accurate eye movements within 2 weeks. Overall, these impressive results are not surprising, as it is well known that the human vestibulo-ocular reflex (VOR) can adapt to extreme misalignment (180 degrees) following continuous wearing of reversing prisms (Gonshor and Jones, 1976). Together, these findings underscore the significance of plasticity within central vestibular pathways in enhancing the effectiveness of prosthetic stimulation.

#### ii. Individual vestibular nerve afferents encode head motion in two directions by modulating around a baseline firing rate

Hair cells within the vestibular periphery are oriented such that head motions in opposite directions result in the increase or decrease of the electrical activity within the cell body, which in turn modulates the rate of the innervating afferent fibers. At rest (e.g. when there is no head motion), primate vestibular afferents display a robust baseline firing rate (i.e. ~100 spikes/s in macaque monkeys; reviewed in Cullen, 2019; Figure 1B, left). During head motion, the firing rate of a given afferent is then modulated relative to this baseline rate depending on the deflection direction of the innervated hair cells (Figure 1B, middle and right). As a result, each vestibular afferent conveys information about head movement in two opposite directions, by either increasing or decreasing firing rate relative to its baseline. In ongoing clinical trials, vestibular prostheses are typically only implanted in one ear to minimize the potential risks and costs as compared to bilateral implantation (e.g. Boutros et al., 2019a; Golub et al., 2014; Guinand et al., 2015). Thus, to generate responses in both directions, mapping strategies typically utilize the same fundamental principle described above. For example, bidirectional sensitivity has been achieved by modulating the stimulation rates about a baseline rate of typically ~100–150 pulses/s (e.g. Boutros et al., 2019a; Dai et al., 2011). Notably, when this baseline is first turned on, it initially causes an imbalance in the input to the vestibular system. Implanted patients and animals thus must first adapt to this applied baseline stimulation prior to receiving head motion-dependent modulation (approximately 30 min in patients [Boutros et al., 2019a], 3 hr in rhesus monkeys [Dai et al., 2013], and <1 day in squirrel monkeys and guinea pigs [Merfeld et al., 2006; Merfeld et al., 2007]).

#### iii. There are two classes of vestibular afferents, and they display distinct response dynamics

**Figure 3.**
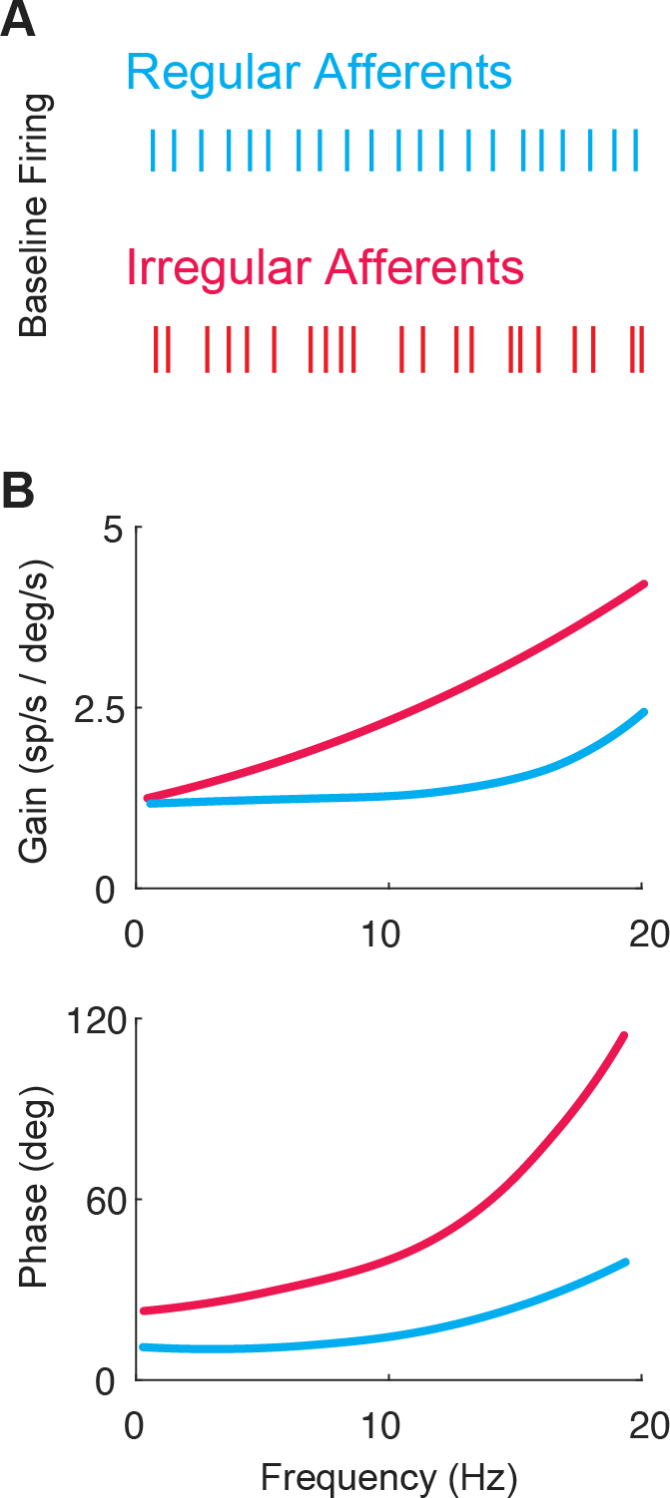
The two classes of vestibular afferents exhibit different baseline firing patterns and responses to head rotations. (**A**) Firing patterns of regular (blue) and irregular (red) afferents at rest. (**B**) While both classes display high-pass tuning, i.e., increasing gain and phase lead as a function motion frequency, irregular afferents show stronger high-pass tuning. Adapted from Figure 4 from Massot et al., 2011.

Vestibular afferents comprise two main classes: regular and irregular afferents. These two classes can be distinguished on the basis of relative differences in resting baseline discharge regularity and morphology (reviewed in Goldberg, 2000). Most notably, as their names suggest, regular afferents fire action potentials at regular intervals at rest while irregular afferents do so with irregular intervals (Figure 3A). In the context of vestibular prosthesis development, it is especially noteworthy that irregular afferents respond more readily to electrical stimulation than regular afferents and thus could be preferentially recruited in response to prosthetic stimulation (Goldberg et al., 1984; Minor and Goldberg, 1991). However, at the relatively high electrical currents typically used for vestibular prostheses stimulation, both afferent types are thought to be recruited (Mitchell et al., 2016; Steinhardt et al., 2022).

The response dynamics of both regular and irregular canal afferents have been well characterized (reviewed in Cullen, 2019; Goldberg et al., 2012). Both regular and irregular afferents display high-pass tuning in their responses to head rotation; their response gains and phase leads increase with increasing stimulus frequency (Figure 3B). Moreover, irregular afferents show stronger high-pass tuning than their regular counterparts. Notably, irregular afferent response gains are greater than those of regular afferents over much of the physiologically relevant range of head motion (Figure 3B, top). In addition, the responses of irregular afferents reach higher phase leads with increasing frequency such that they actually encode angular head acceleration (rather than velocity) at higher frequencies (Figure 3B, bottom). Overall, the high-pass tuning displayed by regular and irregular afferents is well described by linear models at low frequencies and amplitudes of motion (reviewed in Cullen, 2019).

#### iv. Vestibular afferents display significant nonlinearities in response to natural head motion

**Figure 4.**
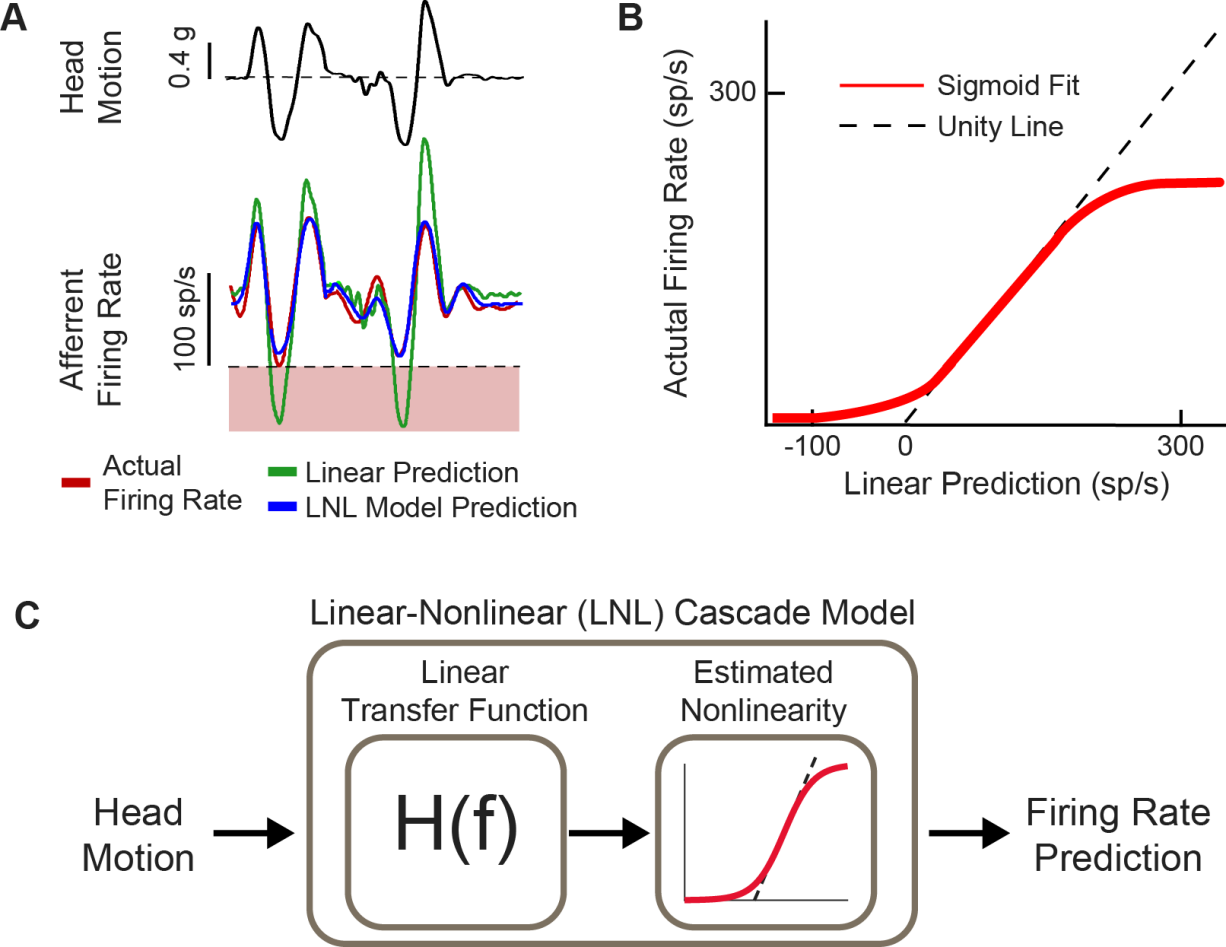
Natural head motion can drive afferent firing to exhibit nonlinearity. (**A**) Afferent firing in response to natural head motion is better predicted by a linear-nonlinear (LNL) model compared to a linear model alone. (**B**) The estimated nonlinearity from afferent firing in (**A**). (**C**) Schematic of the LNL model that can accurately predict afferent firing in response to natural head motion. Panels A–C have been adapted from Figure 4 from Schneider et al., 2015.

A major finding within the last decade is that vestibular stimuli experienced during natural behaviors often exceed both the amplitude and frequency range used in standard laboratory experiments in both humans and animal models (Carriot et al., 2014; Carriot et al., 2017; Schneider et al., 2015). Importantly, such natural stimuli will easily drive the firing of vestibular afferents into cut-off and/or saturation such that they exhibit substantial nonlinearities (Figure 4A and B; Schneider et al., 2015). Accordingly, linear models are not able to describe the responses of afferents across natural behaviors. Instead, a linear-nonlinear cascade model (Figure 4C)—including a nonlinearity that is well approximated by a sigmoidal function—is required to faithfully represent vestibular afferent responses (Mackrous et al., 2022; Schneider et al., 2015).

**Figure 5.**
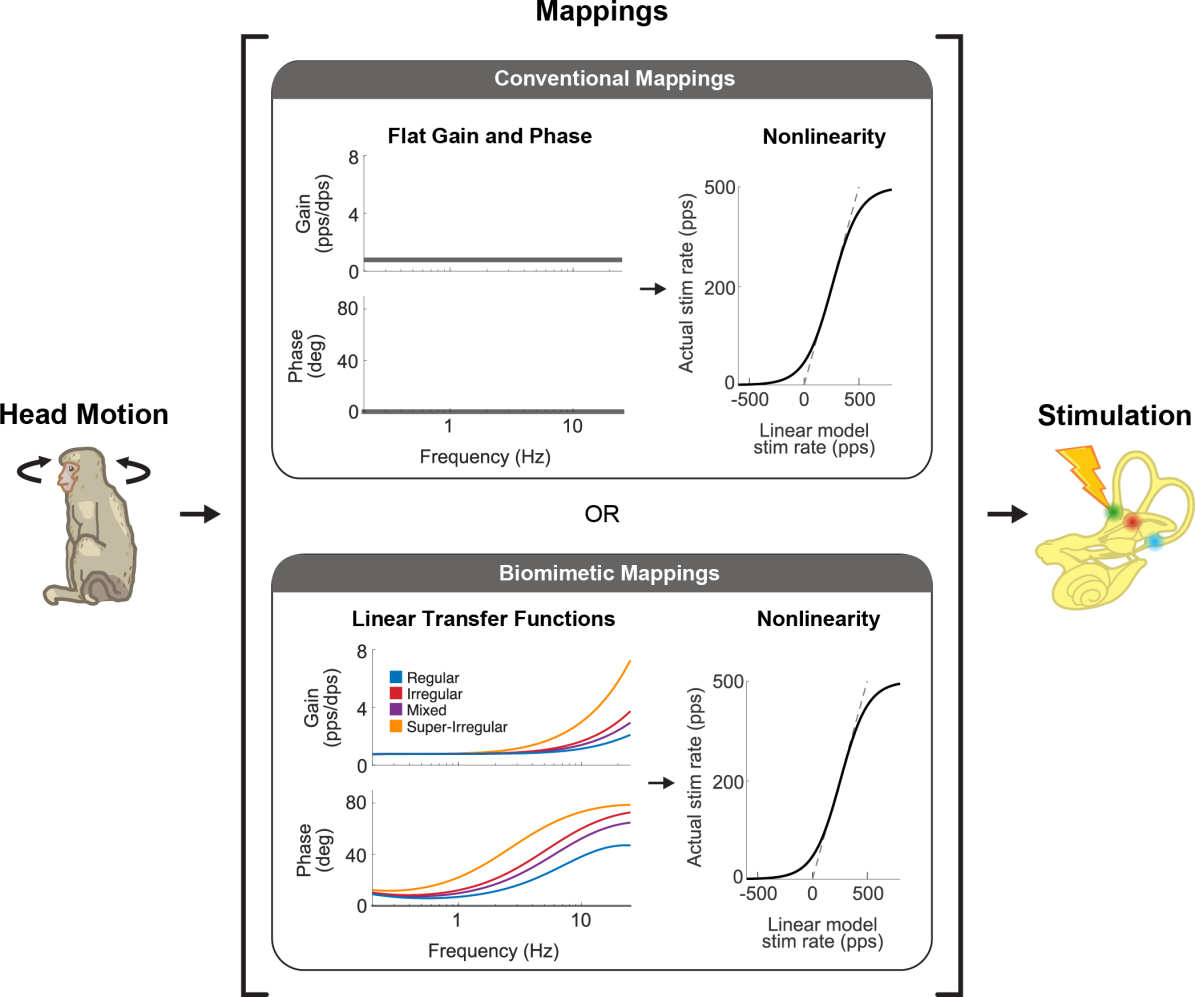
There are two main strategies that vestibular prostheses use to map head motion into the pulse rate of nerve stimulation. Conventional mappings (top) directly link instantaneous head velocity to a specific stimulation rate (i.e. flat gain and no phase lead, only accounting for nonlinearity). In contrast, biomimetic mappings (bottom) use a linear-nonlinear cascade that well captures how vestibular afferents naturally behave. The linear part mimics the response dynamics of the afferents while the nonlinear part mimics the natural cut-off and saturation behaviors. This figure has been adapted from Figure 1 from Wiboonsaksakul et al., 2022.

Implementation of the response dynamics and nonlinearity of vestibular afferent encoding (reviewed above in sections iii and iv) occurs in the mapping of head velocity to pulse rate within the vestibular prosthesis (Figure 5). Generally, these mappings have been programmed to directly link angular head velocity to a specific stimulation rate in the encoding scheme (i.e. constant gain and no phase lead at all frequencies; Figure 5, top; see Boutros et al., 2019a; Chow et al., 2021; Perez Fornos et al., 2014, for examples) or to implement the response dynamics known at the time that only focused on attenuation of responses at low frequencies but not the gain increase at higher frequencies (e.g. Gong and Merfeld, 2000; Gong and Merfeld, 2002). However, these mappings did not account for response dynamics of afferents over much of the frequency range corresponding to natural head motion (i.e. up to 20 Hz; Carriot et al., 2014, Carriot et al., 2017; reviewed in Cullen, 2019). While the nonlinearity is accounted for in all prosthesis mappings (e.g. Boutros et al., 2019a; Perez Fornos et al., 2014), only recently was the complete linear transfer functions of afferent coding implemented into a vestibular prosthesis design (Figure 5, bottom; Wiboonsaksakul et al., 2022). It is noteworthy to point out that these transfer functions are based on neural recordings from rhesus monkeys since the actual responses in humans are not known. However, based on genetic, anatomic, and ethologic similarities, we speculate that neural responses from rhesus monkey should well approximate those of humans.

Finally, recent developments in vestibular prostheses have also made use of stimulation approaches utilizing amplitude modulation rather than (or in addition to) pulse rate modulation. Based on computational modeling of vestibular periphery, it has been proposed that while pulse rate modulation alters the firing rates of recruited afferents, pulse amplitude modulation instead alters the number of afferents that are recruited (Nguyen et al., 2016). Studies measuring eye movements in chinchillas and rhesus monkeys have further suggested that pulse amplitude modulation is at least as effective as pulse rate modulation (chinchilla: Davidovics et al., 2012; monkey: Davidovics et al., 2013; Nie et al., 2013; Phillips et al., 2018) and that co-modulation of stimulation rate and amplitude could potentially extend the dynamic range of the device (Davidovics et al., 2012; Davidovics et al., 2013). One major caveat, however, is that as the current amplitude increases, it spreads to a larger area of tissue and recruits afferents from neighboring canals resulting in response misalignment (Davidovics et al., 2013). In addition, a recent computational study has shown that current amplitude can have direct, nonlinear effects on the activity of single afferents, which could further complicate the firing of the recruited afferents (Steinhardt et al., 2022).

### Central vestibular pathways

**Figure 6.**
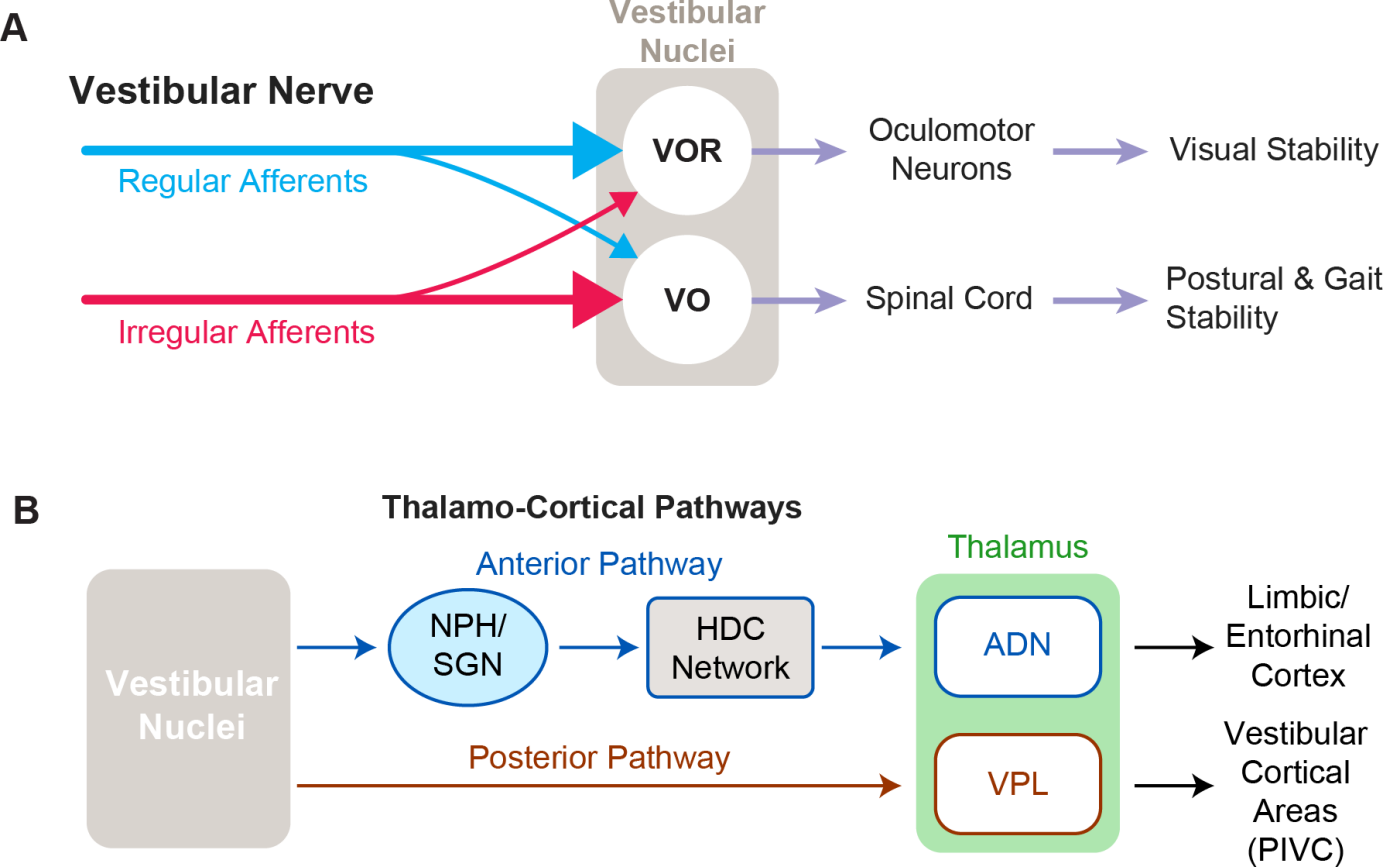
Functional pathways of the vestibular system. (**A**) The two main cell types in vestibular nuclei are vestibulo-ocular reflex (VOR) neurons (top), which are involved in visual stability, and vestibular-only (VO) neurons (bottom), which are responsible for maintaining postural and gait stability. (**B**) Vestibular signals travel through two different pathways to reach the anterior and posterior parts of the thalamus, which are involved in navigation and self-motion perception, respectively.

The goal of vestibular prostheses is to restore vestibular functions that are ultimately mediated by central vestibular pathways. In particular, the vestibulo-motor pathways that generate stabilizing eye and head movements are well characterized and provide easily quantified readouts of prosthesis performance. Vestibular afferents project from the vestibular periphery via the VIII nerve to the brainstem, where they directly synapse with neurons in the vestibular nuclei. Functionally, there are two main cell classes in the vestibular nuclei: those that help maintain gaze stability and those that contribute to postural stability and perception of self-motion (Figure 6; reviewed in Cullen, 2019). The circuits and functional roles of these two classes of central neurons, namely (i) VOR neurons and (ii) vestibular-only (VO) neurons, are each considered below.

VOR neurons are the middle link in the vestibular motor reflex pathways that ensure visual stability via the VOR (Figure 6A, top). This essential reflex provides visual stability during our everyday activities, generating eye movements that are equal in magnitude but opposite in direction to ongoing head movement. VOR neurons receive direct VIII nerve input and in turn project directly to extraocular motor neurons that control the eye muscles, constituting a very fast reflex pathway (~5 ms latency; Huterer and Cullen, 2002). These neurons both respond to vestibular stimulation and are sensitive to eye movements, consistent with their role in gaze stabilization. Although VOR neurons receive direct projections from both afferent types, their primary input is from regular afferents (Boyle et al., 1992; Goldberg et al., 1987; Highstein et al., 1987; Minor and Goldberg, 1991). Overall, the response dynamics of regular afferents are well matched to compensate for the delays within the VOR pathways (i.e. synaptic, neural, and muscle activation time) and the dynamics of the oculomotor plant to produce VOR eye movements that temporally match the time course of the head movement (Boyle et al., 1992; Goldberg et al., 1987; Minor and Goldberg, 1991). In addition, the spiking activities of regular afferents, and consequently VOR neurons, faithfully encode the detailed time course of head motion, a coding feature that is required for the generation of accurate compensatory VOR eye movements (Mackrous et al., 2020). Given that the VOR is highly robust and that VOR eye movements can be readily measured, to date, the vast majority of clinical and animal studies have focused on the VOR as the main behavioral output to quantify the efficacy of vestibular prosthetic stimulation.

VO neurons are the second primary class of neurons in the vestibular nuclei (Figure 6A, bottom). Their name reflects the fact that they respond to vestibular stimulation but are completely insensitive to eye movements. VO neurons serve a vital role in controlling postural stability via the vestibulo-spinal pathway. VO neurons send projections to the spinal cord—predominately via indirect pathways that include relay structures such as the interstitial nucleus of Cajal and reticular formation, as well as direct projections (Boyle et al., 1992; Goldberg et al., 1987; Highstein et al., 1987). In contrast to VOR neurons, VO neurons receive their primary input from irregular rather than regular afferents (Boyle et al., 1992; Goldberg et al., 1987; Highstein et al., 1987). The more dynamic responses of irregular afferents (i.e. larger gains and phase leads) are optimized to generate robust postural responses which must account for the larger inertia of the body relative to the eye (Minor and Goldberg, 1991; Peterson, 1998). Moreover, in contrast to VOR neurons, the majority of VO neurons do not faithfully encode the time course of head movements but instead optimize coding via temporal whitening—a property thought to contribute to ensuring postural control and perceptual stability during everyday activities (Mackrous et al., 2020). Similar to VOR, prosthesis-evoked vestibulo-spinal reflex (VSR) head movements are measured as the behavioral output of prosthetic stimulation (Mitchell et al., 2013; Wiboonsaksakul et al., 2023).

Importantly, in addition to mediating the VSR, VO neurons also make important contributions to ascending vestibular pathways that contribute to perception of self-motion and orientation (Figure 6B). In particular, VO neurons send projections via the posterior vestibulo-thalamic pathway, to the thalamus and cortex (reviewed in Cullen and Taube, 2017). This pathway targets the ventroposterior lateral thalamus of the thalamus, which in turn projects to multiple cortical and subcortical areas, including the parieto-insular vestibular cortex, which are thought to be involved in self-motion perception (reviewed in Cullen and Chacron, 2023). In contrast, the vestibular nuclei also send projections via the nucleus prepositus (McCrea et al., 1987) and subsequent relay nuclei to anterodorsal nucleus of the thalamus. In turn, the anterior vestibulothalamic pathway provides vestibular information to the head direction cell network, which contributes to regions involved in navigation and spatial memory including the retrosplenial cortex, entorhinal cortex, and the hippocampus (reviewed in Cullen and Taube, 2017).

While the VOR and VSR, as well as the vestibulo-thalamic pathways, receive differential input weighting from regular and irregular afferents, current vestibular prostheses deliver a single stimulation pulse (with large current amplitude) to recruit all afferents within the target semicircular canal. Consequently, prosthetic stimulation, unlike natural stimulation, is likely to evoke similar activation of both regular and irregular afferents during a specific head motion. To restore activities of both regular and irregular afferents to their (different) natural behaviors, the prosthesis must theoretically be able to independently stimulate each afferent group. While it has been proposed that such a directed approach could potentially be achieved by leveraging the lower recruitment threshold of irregular afferents (i.e. Wall et al., 2003), we speculate that it would be difficult to obtain precise afferent selectivity using currently available pulsatile stimulation-based technology.

## Current functional outcomes and limitations

Since the conception of the vestibular prosthesis and initial pioneering animal studies in the early 2000s (e.g. Gong and Merfeld, 2000; Gong and Merfeld, 2002, see also Wall et al., 2003, for a review), promising results reported in follow-up animal studies have led to the development of three human prosthesis prototypes which are currently in ongoing clinical trials. Currently, the criteria for implantation in these clinical trials include unsteadiness when walking or standing accompanied by blurred vision or oscillopsia during head movements and/or worsening unsteadiness in the dark or on uneven ground (van de Berg et al., 2020). Vestibular prostheses are thus aimed at patients who cannot compensate centrally and are still experiencing permanent disability due to vestibular loss. Notably, across ongoing clinical trials, two primary strategies are used to modulate vestibular afferent activity. The first strategy is to directly stimulate afferents such that their activity is modulated by head motion similar to healthy individuals (Geneva-Maastricht research group and Johns Hopkins University research group; e.g. Boutros et al., 2019a; Guinand et al., 2015). As discussed above, within this framework it is possible to independently vary or co-vary pulse rate and pulse amplitude modulation to optimize afferent recruitment and stimulation coupled to head motion. A second strategy is to modulate afferent firing such that noisy or inconsistently generated signals are overridden by the prosthesis (University of Washington research group; e.g. Golub et al., 2014). It has been proposed that this strategy, termed a ‘vestibular pacemaker’, is of most use to patients with Meniere’s disease (Golub et al., 2014; see also Wall et al., 2003 and Merfeld et al., 2006, for original conception and animal prototype results). Below, we discuss the functional restorations brought about by both the foundational studies in animal models and clinical trials in patients, particularly those utilizing a head movement coupled modulation strategy.

### VOR and visual stability

**Figure 7.**
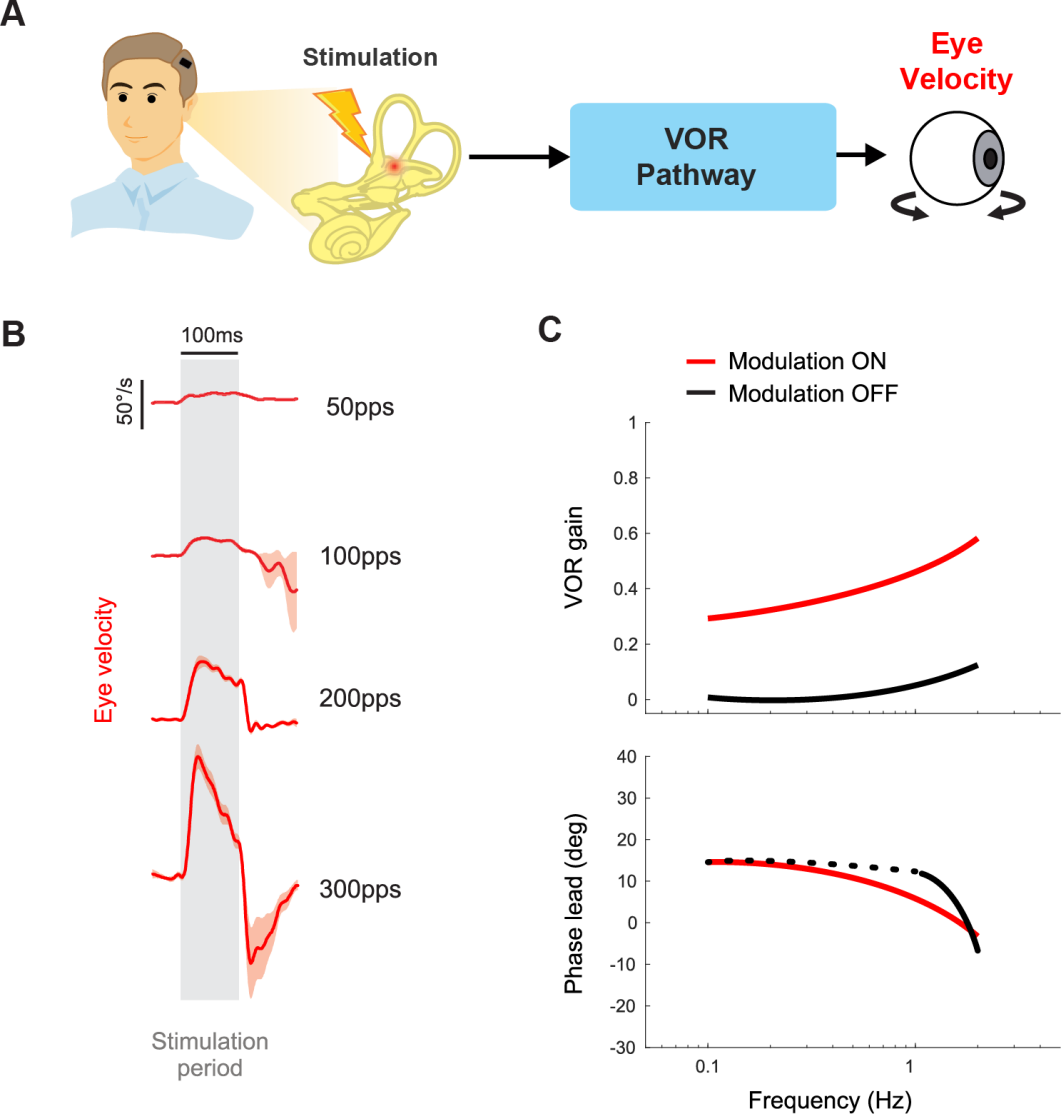
Vestibular prostheses can restore vestibulo-ocular reflex (VOR) functions to improve visual stability. (**A**) Pathway diagram of the VOR. Head motion information is encoded in prosthetic stimulation pulses to vestibular nerve. This information, in turn, generates eye movement that counteracts the head movement. (**B**) Prosthetic pulse trains can evoke VOR eye movement associated with the specific canal being stimulated. Adapted from Figure 1 from Mitchell et al., 2013. (**C**) In normal operation where the prosthetic pulses encode the head motion, gain and phase of the VOR response can be calculated. Data from a human clinical trial shows improved VOR gains when prosthesis modulation is on. Adapted from Figure 8 from Boutros et al., 2019a.

As mentioned above, over the past decades of vestibular prosthesis development, the VOR has been extensively used to quantify performance (i.e. Figure 7A and B). In healthy animals and human participants, the VOR eye movements are generally quantified by calculating their gain and phase in response to sinusoidal head rotations across a range of frequencies. In healthy humans, the gain of the rotational VOR is ~1.0, meaning there is a compensatory eye velocity equal to the head velocity and in the opposite direction. Accordingly, to facilitate comparison with normal VOR function, studies of implanted nonhuman primates (Boutros et al., 2019b; Dai et al., 2011) as well as implanted patients (Boutros et al., 2019a; Perez Fornos et al., 2014) have measured the eye movements evoked by sinusoidal head rotations. In general, the VOR gain shows significant improvements when the prosthesis modulation is on vs. off (Figure 7C). This is accompanied by improved visual acuity during treadmill walking and head impulses when the prosthesis is turned on Ayiotis et al., 2022; Guinand et al., 2016; Starkov et al., 2020. Additionally, while oscillopsia is not directly correlated with VOR gain across unilateral vestibular loss, bilateral vestibular loss, and cerebellar patient populations (Palla et al., 2008; Geisinger et al., 2024; Morland et al., 1998; Grunfeld et al., 2000), patients in both the Johns Hopkins and Geneva-Maastricht clinical trials have reported reduced or absent oscillopsia symptoms when the prosthesis is turned on Ayiotis et al., 2024; Boutros et al., 2019a; Chow et al., 2021; van Stiphout et al., 2022. Taken together, these findings suggest that the prosthesis has significant potential to improve visual stability.

However, there remains significant room for improvement in evoking sufficient VOR via vestibular prostheses. The gains of the evoked VOR eye movements are not fully compensatory (i.e. gain<1; Figure 7C). Notably, there is considerable variability across both implanted nonhuman primates (Wiboonsaksakul et al., 2022) and patients (Boutros et al., 2019a), such that VOR gains typically range between ~0.2 and 0.6. One obvious approach to improve the VOR gain is to alter the mapping scheme between the detected head motion and stimulation modulation, such that the same head motion gives rise to greater modulation, e.g., by increasing the gain in Figure 5 (Perez Fornos et al., 2014). However, a disadvantage of this strategy is that it comes at the cost of a reduction in the dynamic range that the prosthesis can encode before entering cut-off/saturation. Fortunately, congruent extra-vestibular signals (i.e. visual, tactile, proprioception) can augment prosthetic stimulation to improve the VOR gain. For example, recent work in nonhuman primates has demonstrated greater VOR gain (though still not fully compensatory) from prosthetic stimulation in conjunction with physical rotation compared to prosthetic stimulation alone (Dai et al., 2012; Wiboonsaksakul et al., 2022). The effect is supra-additive, such that the gain during the combined condition was more than the sum of the gains in each separate condition alone.

### Balance and gait

A classic study by Suzuki and Cohen, 1964, demonstrated that electrical stimulation of the afferents evoked stereotyped postural reflexes in the neck and limbs, in addition to compensatory VOR eye movements. However, measuring and quantifying these evoked compensatory postural responses is inherently more complex relative to those of the evoked VOR. As a result, studies of the effects of vestibular prostheses on posture and balance are at a more nascent stage. One essential class of vestibular postural reflexes—the vestibulocollic reflex—moves the head on the body to stabilize the head in space as the body moves under it (reviewed in Goldberg and Cullen, 2011). Short pulse trains applied via the vestibular prostheses evokes not only VOR eye movements, but also head motion contralateral to the stimulated side in implanted monkeys (Mitchell et al., 2013). Notably, the amplitude of the evoked compensatory head movement increases with stimulation current amplitude and pulse rate (Mitchell et al., 2013), and the activation of the neck muscles driving these compensatory head movements has been measured via EMG in patients (Fornos et al., 2019).

**Figure 8.**
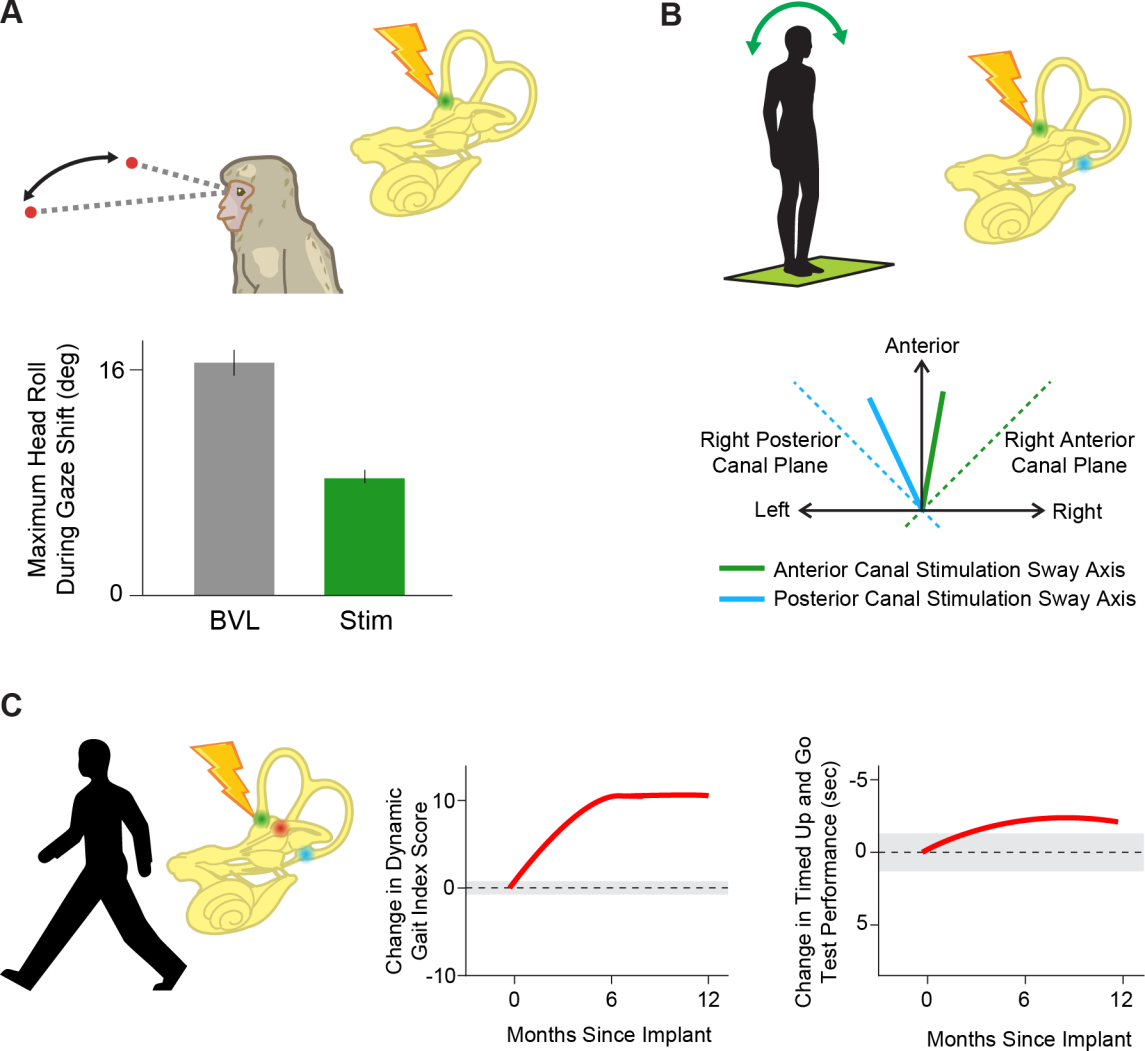
Prosthetic stimulation can drive reflexes and help restore balance. (**A**) Providing head velocity information in the anterior canal plane through a prosthesis during gaze shifts in a rhesus macaque reduces overall head motion. Adapted from Figure 6 from Thompson et al., 2016. (**B**) Stimulating the vertical (anterior, green and posterior, blue) semicircular canals individually in humans drives postural sway (solid lines) in the direction of that canal’s plane (dotted lines). Adapted from Figure 3 from Phillips et al., 2013. (**C**) Stimulating all three semicircular canals independently with a prosthesis leads to improved scores on dynamic gait index and timed up and go tests, important clinical tests of posture and gait during everyday activities. Adapted from Figure 2 from Chow et al., 2021.

The second essential class of vestibular postural reflexes are those which generate limb movements to stabilize the whole body in response to a head motion perturbation. Experiments in implanted monkeys have shown improvements in posture when stimulation is provided by a vestibular prosthesis encoding head motion via pulse rate modulation during gaze shifts (Figure 8A; Thompson et al., 2016). Moreover, in implanted patients, short pulse trains applied via the vestibular prostheses to each semicircular canal can generate whole-body postural responses that are biased toward the stimulated canal’s plane (Figure 8B; Phillips et al., 2013). Similarly, more recent studies in implanted patients have demonstrated that modulated vestibular prosthetic stimulation improves performance on both tests that directly assess vestibular function, such as the modified Romberg, and more general tests of posture and balance, including the timed walk and timed up and go tests (Figure 8C; Chow et al., 2021). While the latter two tests are not exclusively vestibular-dependent, the improvements in these tasks underscore the potential of vestibular prosthetic stimulation to enhance overall balance and posture in daily life.

### Higher-order vestibular functions

#### Self-motion perception

To date, objective quantification of how vestibular prosthetic stimulation alters the perception of self-motion in clinical settings has not been directly conducted, in contrast to the often-quantified VOR. However, it has been reported that, at the onset of baseline prosthetic stimulation, implanted patients initially experience vestibular percepts (i.e. feelings of being rotated about the axis associated with the stimulated canal), that markedly attenuate within minutes (e.g. Boutros et al., 2019a; Crétallaz et al., 2020; Guinand et al., 2015). We speculate that this percept is due to activation of posterior thalamocortical vestibular pathways, including the inter- and intra-hemispheric cortical networks (Calzolari et al., 2021; Hadi et al., 2022; Hadi et al., 2024), responsible for self-motion perception in normal individuals (also reviewed in Cullen and Chacron, 2023; Cullen and Taube, 2017). How motion-modulated stimulation affects these pathways and the resulting perception (e.g. change in perceptual threshold) remains an open question that would benefit from future study.

#### Spatial orientation

Two laboratory studies in rhesus monkey objectively quantified the effect of vestibular prosthetic stimulation on spatial orientation. In these experiments, monkeys were trained to align a light bar with gravity (i.e. earth vertical), either during static or continuous roll tilt. In both cases, when tilts were accompanied by consequent prosthetic stimulation, the monkeys’ reports of earth vertical were biased toward the direction of the stimulated canal (Karmali et al., 2021; Lewis et al., 2013a). Thus, these studies provide evidence that in addition to improving vestibular motor functions, vestibular prostheses can also likely improve the perception of spatial orientation in patients.

#### Navigation and memory

Through their projections to cortical and subcortical areas, vestibular pathways also contribute to a variety of other higher-order functions, including navigation and spatial memory in humans (reviewed in Zwergal et al., 2024). To date, the effect of prosthetic stimulation on navigation and memory has not yet been directly tested. However, it is likely that the reported improvements in quality of life (Chow et al., 2021; van Stiphout et al., 2022) are in part due to improvement of these functions. Furthermore, we anticipate that since noninvasive activation of the vestibular afferents via galvanic vestibular stimulation can increase activity in vestibular-sensitive subcortical and cortical areas even following bilateral vestibular loss (Helmchen et al., 2020; Ruehl et al., 2022; Stephan et al., 2005), future work is likely to demonstrate that vestibular prostheses likewise stimulate these areas and can in turn produce improvements in navigation and spatial memory.

### Summary and future directions

Functional outcomes from animal studies and clinical trials highlight promising advancements in the development of a vestibular prostheses. However, most studies rely on VOR gains as a performance metric, which, though integral to the development of vestibular prostheses, do not always correlate with broader vestibular functions. For instance, prior studies have reported the uncoupling of VOR function from self-motion perception in trained dancers (Nigmatullina et al., 2015). VOR gains also poorly predict oscillopsia (Grunfeld et al., 2000) and fall risk (Dobbels et al., 2020) in patients with bilateral vestibular loss. Furthermore, individuals with vestibular implants can exhibit significant improvements in balance and gait despite suboptimal VOR gains (Chow et al., 2021). These findings underscore the need to assess higher-order vestibular functions, such as self-motion perception and fall risk, in prosthesis optimization. Additionally, current implantation criteria do not explicitly exclude patients based on co-morbidities, except those that would preclude surgery (van de Berg et al., 2020). However, evidence suggests chronic conditions, such as diabetes, worsen self-motion perception thresholds, postural stability, and fall risk over time (La Scaleia et al., 2024). Incorporating co-morbidity assessments into patient selection criteria could significantly enhance future clinical outcomes.

## Opening the ‘black box’: future directions informed by neurophysiology

Systems neuroscience is at an exciting juncture with the standard practice shifting toward recording extensive datasets from neural ensembles, a promising development relative to the traditional single-cell recording approach. This shift will further advance our ability to fill a critical gap in our knowledge and answer the fundamental question: How does the brain respond to vestibular prosthetic stimulation? Most studies to date have treated the brain as a ‘black box’ within an input-output system, by applying different input stimulation protocols and then measuring changes in behavioral responses as the output. Such approaches have proven useful in the nascent stage of technology; however, they cannot provide insight into the neural computations that give rise to prosthesis-evoked behaviors. Only by directly studying neural circuits at the population level can we identify the impediments in neural processing affecting neuroprosthesis performance to innovate solutions. Thus, advancing the next generation of implants hinges on experiments focused on understanding how the brain processes prosthetic vestibular inputs in comparison to normal processing in healthy animals. Fortunately, the recent advancement in high-density recording technology should allow for more studies, both at the single-neuron and population level, that further our understanding of neural processing of prosthetic inputs. Accordingly, in this section, we will consider recent studies at the level of single neurons that have provided important new insights, as well as the avenues currently being explored for improving vestibular implants.

### Plasticity in stimulated pathways: advantage or challenge?

**Figure 9.**
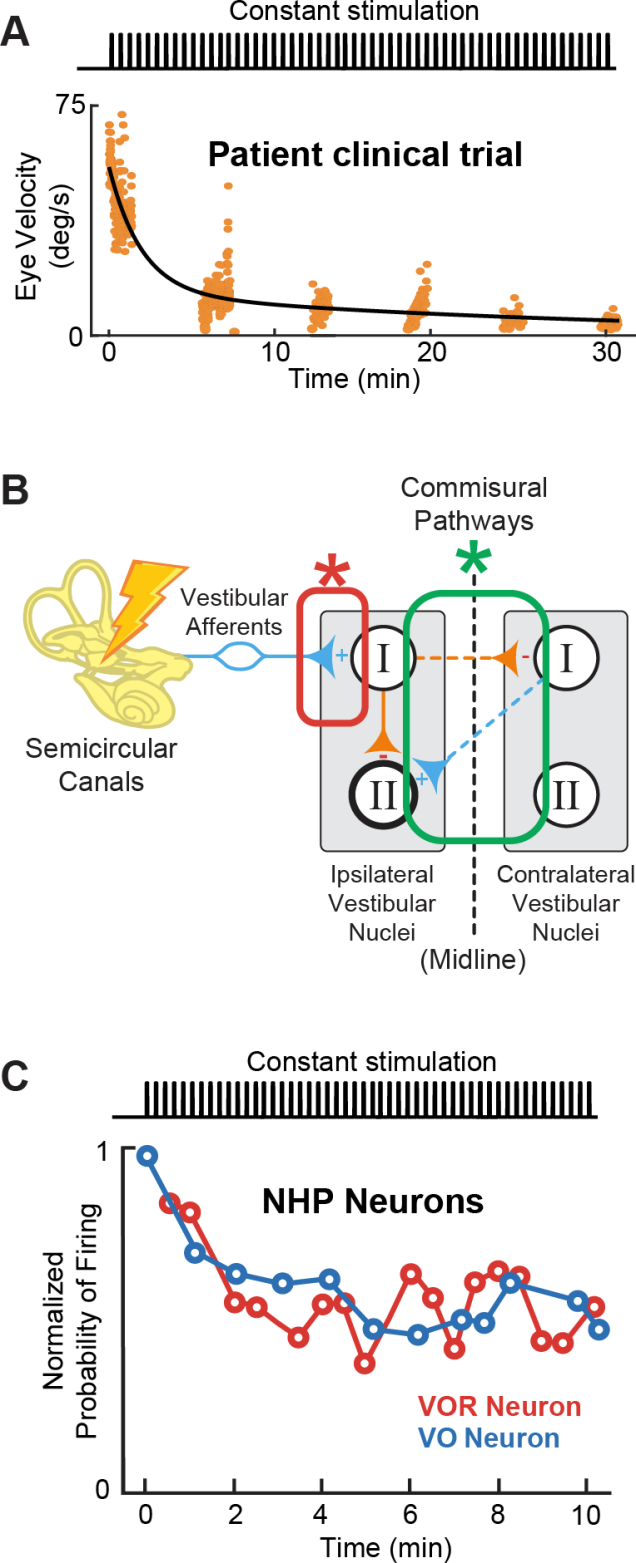
Adaptation to prosthetic pulses at the afferent-vestibular nuclei synapses is problematic. (**A**) Vestibulo-ocular reflex (VOR) adaptation to onset of baseline prosthetic stimulation from a clinical trial. Adapted from Figure 5 from Boutros et al., 2019a. (**B**) Compensatory synaptic changes occur at the afferent-vestibular nuclei synapses (red) as well as in central commissural pathways (green). (**C**) Problematic adaptation of VOR and VO neurons to prosthetic stimulation. Adapted from Figure 6 from Mitchell et al., 2016; Mitchell et al., 2017.

In clinical trials of vestibular prostheses, the onset of baseline stimulation initially induces an asymmetry in vestibular input between the left and right ears that is interpreted as a head movement, and thus causes nystagmus and postural instability (Boutros et al., 2019a; Chow et al., 2021; Lewis et al., 2013b). Patients then adapt to this unilateral baseline stimulation rate over a relatively rapid time course (Figure 9A). It has generally been assumed that adaptation to this asymmetric vestibular input is mediated by central pathways, notably the commissural vestibular pathways (Figure 9B, green; Chiang et al., 2011; Della Santina et al., 2007; Merfeld et al., 2006; Merfeld et al., 2007). Indeed, experiments in rats and mice suggest plasticity in commissural pathways along with cerebellar and cortical vestibular areas that produces changes in functional connectivity within minutes to hours following unilateral vestibular loss, a condition similar to the onset of baseline stimulation with a vestibular prosthesis, which induces asymmetry in the input to central vestibular pathways (Cirelli et al., 1996; Grosch et al., 2021; Kai et al., 2022; Kitahara et al., 1997; Zwergal et al., 2016). In this view, adaptation is beneficial and results in a new setpoint, from which a unilateral implant can up- or down-modulate to encode head motion in two opposing directions.

Importantly however, recent studies exploring the effect of prosthetic stimulation on central pathways have instead established that adaptation to prosthetic stimulation is mediated earlier in the pathway, at the afferent-vestibular nuclei synapse (Figure 9B, red; Mitchell et al., 2016; Mitchell et al., 2017). Specifically, experiments in implanted monkeys have revealed that prosthetic baseline stimulation actually leads to the rapid and marked (>50%) reduction in efficacy of the afferent-vestibular nuclei synapse, which in turn substantially reduces efficacy of central vestibular pathways within minutes after stimulation onset (Figure 9C; Mitchell et al., 2016; Mitchell et al., 2017). Adaptation at this synapse is fundamentally problematic because, instead of resulting in a new setpoint (as for central adaptation), it attenuates all prosthetic inputs, including motion-modulated stimulation encoding the head movement information that the prosthesis is trying to restore to the brain. This maladaptive prosthesis-induced reduction in synaptic efficacy occurs at the level of afferent input to both the vestibular nuclei neurons that generate the VOR (Mitchell et al., 2016) and those that control VSR (Mitchell et al., 2017). Rapid complementary plasticity within inhibitory commissural nuclei network contributes to lessen this maladaptive effect of stimulation. However, this rapid plasticity only partially improves behavioral VOR and VSR performance (Mitchell et al., 2016; Mitchell et al., 2017). Critically, only a few minutes of baseline prosthetic stimulation causes reductions in both VOR and VSR behavioral responses that can persist for more than 8 hr in absence of head motion or further prosthetic stimulation (Mitchell et al., 2016; Mitchell et al., 2017). Overall, we speculate that the maladaptive reduction in the efficacy of the afferent-vestibular nuclei synapse is a key reason why implanted patients show incomplete restoration of function (Boutros et al., 2019a). How to effectively reduce this undesirable early adaptation and what stimulation parameters—i.e., current amplitude (at sub- or peri-threshold levels) and pulse shapes—contribute to this maladaptation remain open questions for future investigations.

### Strategies for desynchronizing afferent activity

The adaptation at the afferent-vestibular nuclei synapse described above is fundamentally problematic. This is because it will not only attenuate central neuron responses to baseline prosthetic stimulation but also to head motion modulated stimulation. As a result, this maladaptation reduces the efficacy of head motion information transfer to the reflex pathways as well as those that contribute to self-motion perception (Figure 1A). This reduction in efficacy is likely due, at least in part, to the synchrony that is induced across vestibular afferents by prosthetic stimulation. The spiking activity of stimulated afferents synchronizes with prosthetic-driven pulse trains because their action potentials are tightly linked to the applied pulses (Mitchell et al., 2016; Steinhardt et al., 2022). Importantly, this synchrony contrasts with what is observed across afferent population responses to natural head movement stimuli (Dale and Cullen, 2013; Yu et al., 2014). In vitro studies have demonstrated that repeated pulsatile stimulation of vestibular afferents induces long-term depression at the afferent-vestibular nuclei synapse (McElvain et al., 2010), thereby providing a mechanism for this observed reduction in central pathway efficacy (Mitchell et al., 2016; Mitchell et al., 2017). Correspondingly, it has been established that cochlear implant stimulation induces artificial synchrony in the firing of auditory afferents (Babalian et al., 2003; Hu et al., 2010; Sachs et al., 1983; Wu et al., 2016) as well as their target neurons in the cochlear nucleus (Babalian et al., 2003). Furthermore, as in the vestibular system, the synchrony induced by cochlear implant stimulation reduces the efficacy of central auditory pathways over time (Hu et al., 2010; Runge et al., 2018).

Ultimately, overcoming the maladaptive effect of vestibular prosthesis induced afferent synchrony will be essential for improving these devices. One general strategy that has been used for cochlear implants is high-frequency subthreshold stimulation. In this strategy, stimulation is delivered at a frequency well beyond that of the afferents’ maximum firing rate, typically 5 kHz (e.g. Babalian et al., 2003; Litvak et al., 2003). Two main approaches have been used to deliver high-frequency stimulus. The first uses a high-frequency as the primary carrier for amplitude-modulated stimulus pulses (Babalian et al., 2003; Hu et al., 2010; Rubinstein et al., 1999), while the second adds a high-frequency, subthreshold ‘buzz’ on top of lower-frequency modulated stimuli (Litvak et al., 2003). The goal of both approaches is to exploit the afferents’ fixed refractory period to produce more variability in response timing across afferents. A second strategy that has been successful in improving the performance of cochlear implants is the use of altered pulse waveforms, most notably delayed pseudomonophasic (DPM) asymmetric stimulus pulses. In particular, improvements in auditory thresholds have been reported for DPM stimulation characterized by a brief, high-amplitude cathodic phase followed by a long, low-amplitude anodic phase (Quass et al., 2020; van Wieringen et al., 2008). We speculate that these improvements are also mediated via a reduction in synchrony and that combining these two general approaches (i.e. high-frequency subthreshold stimulation methods and novel pulse shapes) will further reduce the overall synchrony of vestibular afferent responses, in turn improving vestibular prosthesis performance.

### Physiological implications of hair cell loss for restoring function

Finally, it is noteworthy that both vestibular prostheses and cochlear implants are designed to directly stimulate afferents in their respective systems, bypassing the hair cells of the sensory epithelium which are no longer functional (reviewed in Shibata et al., 2011). Thus, these devices operate on the assumption that their respective afferents remain fundamentally healthy. However, neurophysiological studies of the auditory system have shown that hair cells provide both neurotrophic support and excitatory inputs to afferents. Accordingly, the loss of auditory hair cells not only significantly reduces the sensitivity of afferents fibers (e.g. Hartmann et al., 1984; Kiang and Moxon, 1972; Suthakar and Liberman, 2021) but also results in afferents fiber demyelination and ultimately loss of afferent fiber and their somas within the spiral ganglia over months to years (Kurioka et al., 2016; Nadol, 1997; Spoendlin, 1975; Webster and Webster, 1981). Likewise, work to date focusing on changes in the vestibular periphery following hair cell loss has reported a similar reduction in vestibular afferent somas (Ishiyama et al., 2004). However, promisingly for vestibular as well as cochlear implantation, electrical stimulation of auditory afferents via a cochlear prosthesis has been shown to protect auditory afferents from further degeneration (reviewed in Shibata et al., 2011). Thus, we speculate that implanting as early as possible after vestibular hair cell loss may help improve patient outcomes. Furthermore, a recent study in monkeys found that vestibular implants can effectively evoke eye movements, even when the prosthesis was implanted 10 years after bilateral labyrinthectomy, demonstrating that vestibular implants are likely to be effective even if some afferents have been lost (Wiboonsaksakul et al., 2023). An important area of research moving forward will be to improve our understanding of how to optimally preserve healthy afferent function following the loss of hair cells in both systems (reviewed in Shibata et al., 2011).

## Toward naturalistic stimulation

In conjunction with the efforts to understand how the brain processes prosthetic stimulation, much of the recent developments in vestibular prostheses is toward delivering more naturalistic stimulation back to the nervous system. As in other neuroprostheses, it is widely believed that the brain can more promptly and effectively utilize biomimetic and naturalistic sensory information (reviewed in Iskarous and Thakor, 2019; Saal and Bensmaia, 2015). Below, we review emerging approaches to deliver more naturalistic stimuli via the vestibular prosthesis including the biomimetic encoding of head motion, the addition of otolith organ stimulation, and alternative ways to modulate afferent activity.

### Biomimetic stimulus design

Vestibular prosthesis development has been largely guided by our fundamental knowledge of the anatomy and physiology of vestibular afferents. Yet, as reviewed above, the vast majority of both clinical trials and animal studies have employed mappings that did not consider how vestibular afferents respond during natural everyday activities, or were implemented based on the limited knowledge available at the time. In general, while prior studies have accounted for the fact that afferents modulate their firing rates around a resting baseline and show cut-off/saturation nonlinearities (see Figure 5, top), they did not account for the high-pass response dynamics of the afferents (i.e. increasing gain and phase lead as a function of frequency; Figure 3B). Instead, the prevailing approach in ongoing clinical trials has been to simply map instantaneous head velocity to a specific pulse rate (i.e. flat gain and no phase lead). Because natural head motion can reach frequencies as high as 20 Hz (Carriot et al., 2014; Carriot et al., 2017), it is essential to consider the high-pass dynamics of vestibular afferents. For example, the phase of VOR eye movements evoked by head motion remains compensatory across the physiological range of motion even at frequencies exceeding 20 Hz (Huterer and Cullen, 2002; Ramachandran and Lisberger, 2005). In theory, without the phase compensation provided by the high-pass dynamics of afferents, VOR eye movements would increasingly lag head motion reaching a lag of >50 degrees for higher frequencies within physiological range. This is because an increase in afferent phase lead as a function of frequency is required to compensate for the fixed delays in the VOR pathways (e.g. synaptic transmission and muscle activation, see Discussion in Huterer and Cullen, 2002).

**Figure 10.**
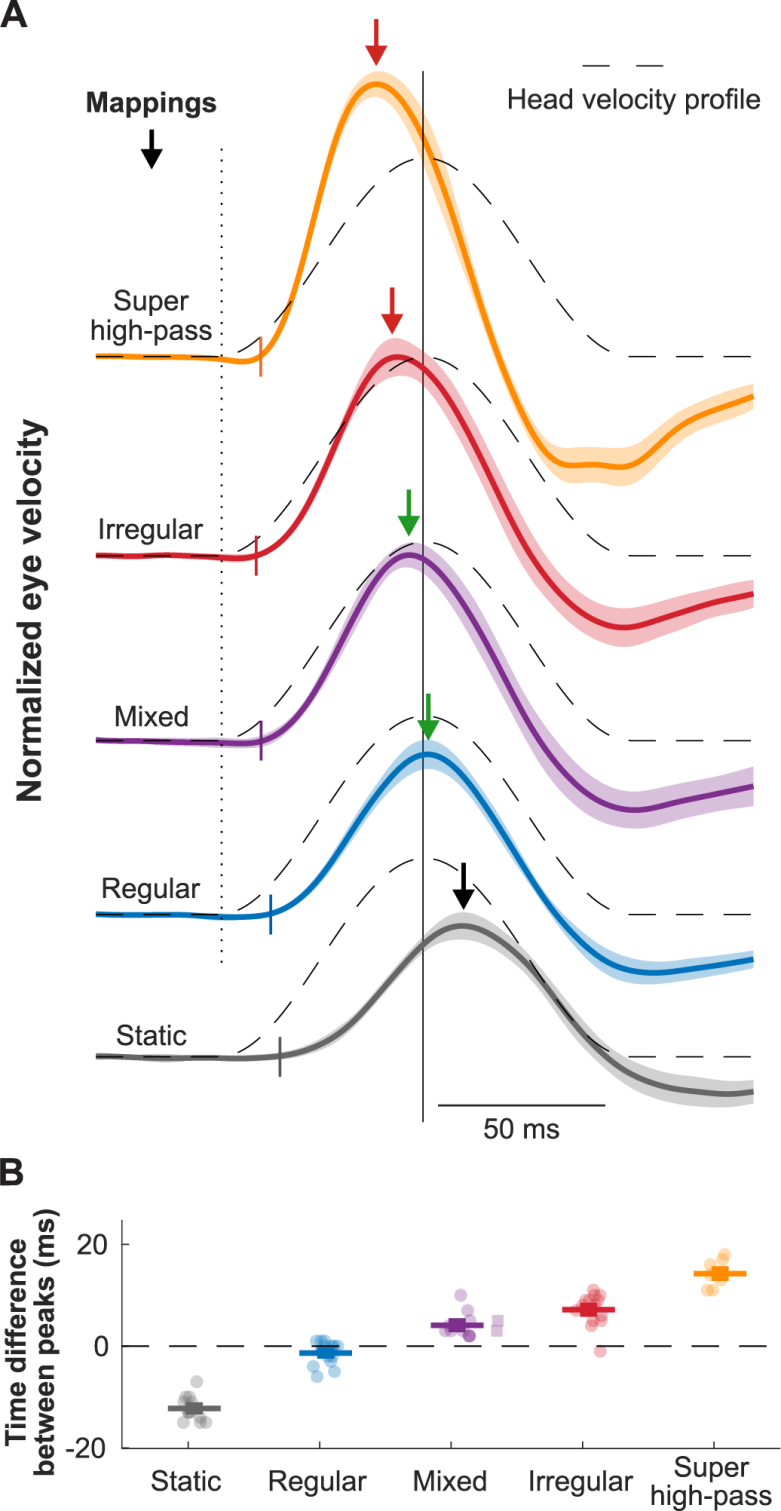
Biomimetic encoding of head motion improves timing accuracy of vestibulo-ocular reflex (VOR). (**A**) Normalized traces of the evoked eye movements during transient head movement using different mappings. Dashed lines show the inverted head velocity. Dotted vertical line denotes the start of the head movement. Solid vertical line denotes the peak of the inverted head movement. Short colored vertical lines indicate the estimated latency. Arrows show the peak of the eye movement response. (**B**) Quantification of the difference between the peak timing of the head and eye velocity for the traces in (**A**). This figure has been adapted from Figure 4 from Wiboonsaksakul et al., 2022.

Indeed, a recent study has confirmed the theoretical scenario above (Wiboonsaksakul et al., 2022). Conventional mappings that did not account for high-pass dynamics of vestibular afferents resulted in decreasing gain and increasingly lagging phase of the evoked VOR, while biomimetic mappings that incorporated high-pass tuning resulted in more robust gain and more accurate phase of the evoked VOR (Figure 5, bottom; Wiboonsaksakul et al., 2022). Further exploration of the parameter space revealed that more extreme tunings were not beneficial in that they produced saturation and unnatural phase advances. Moreover, mapping strategies biased toward the response dynamics of regular afferents yielded better VOR performance than a mapping strategy based on the response dynamics of irregular afferents (Figure 10, blue and purple vs. red). This finding is consistent with prior experimental findings demonstrating that regular afferents make a more significant contribution to VOR pathway than do their irregular counterparts (Boyle et al., 1992; Goldberg et al., 1987; Highstein et al., 1987; Minor and Goldberg, 1991). Future work in vestibular prosthesis development should focus on the relative improvements in performance produced by different biomimetic mappings for different vestibular motor pathways (e.g. regular mapping for VOR vs. irregular mapping for postural control and perception).

### Otolith stimulation

To date all but a handful of studies have focused on vestibular prostheses that aim to restore semicircular canals rather than otolith function. As described below, this semicircular canal focused approach is currently well warranted because of the technical challenges of implanting an otolith prosthesis. However, since natural head movements generally comprises both rotational and linear motion, inputs from both the semicircular canal and otolith are typically processed simultaneously and integrated for both behavior and perception (reviewed in Angelaki and Cullen, 2008). Thus, an important future direction for improving functional outcomes in patients with bilateral vestibular loss is restoring otolith as well as semicircular canal input. The otolith organs, however, are a more challenging target for prosthesis development. Notably, although it is straightforward to activate semicircular canal hair cells in a directionally precise manner (i.e. Figure 2), this is not the case for the utricle and saccule.

**Figure 11.**
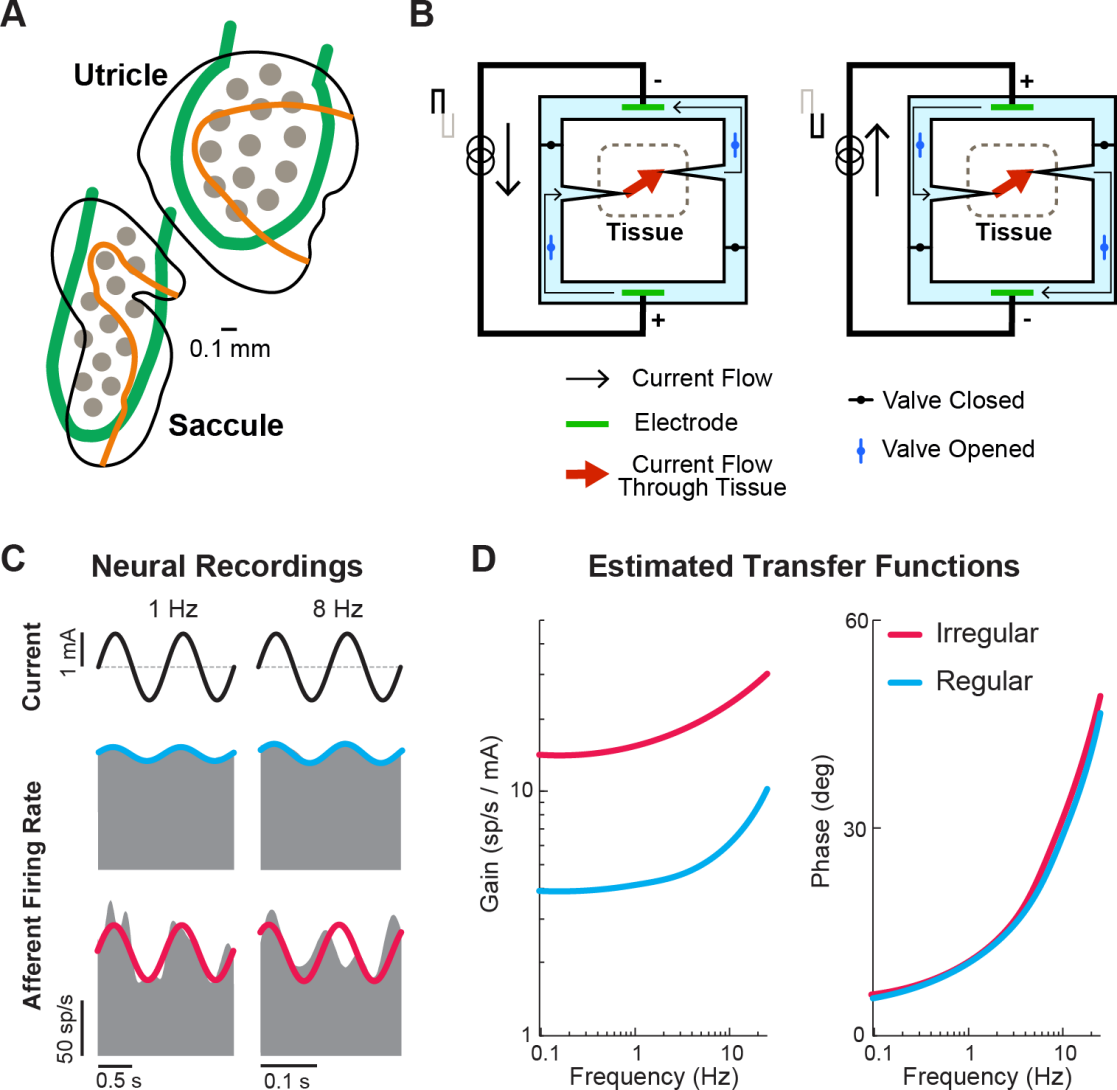
Ongoing developments toward more naturalistic stimulation in vestibular prostheses. (**A**) Schematic of the electrode array for stimulation of the otolith organs. Gray circles denote electrode contacts. Orange lines denote the line of polarity reversal (i.e. where different hair cells are sensitive to different motion directions). Adapted from Figure 1 from Hageman et al., 2020. (**B**) Schematic of a safe direct current stimulator, which can continuously deliver direct current in one direction to tissue while preventing unwanted electro-chemical reactions at the electrode interface. Adapted from Figure 1 from Aplin and Fridman, 2019. (**C**) Firing rate of recorded canal afferents in response to galvanic vestibular stimulation (GVS). (**D**) Estimated transfer functions of the afferent response to GVS from the neural data in (**C**). Panels C and D have been adapted from Figure 2 from Kwan et al., 2019.

A key feature of the otoliths is that their hair cells are distributed over the neuroepithelia surface in a systematic manner that comprises a directional tuning map, with the axis of greatest sensitivity perpendicular to the curved line across the otolith organs (Figure 11A, orange lines). This hair cell alignment allows the utricle and saccule to sense linear motion in the horizontal and vertical plane, respectively, and together detect linear forces in three dimensions. For any given head motion direction, different hair cells are excited or inhibited to many varying degrees depending on how their axis of sensitivity aligns with the head motion. Consequently, stimulating the otoliths in a directionally specific manner requires a great number of electrodes that can selectively and independently stimulate a very focused area of the organ. Despite these challenges, the Della Santina laboratory has recently implanted a multichannel planar stimulating array in the otoliths of chinchillas (Figure 11A, gray circles). Stimulation of the otoliths using the implant successfully generated torsional and vertical eye movements consistent with vestibulo-ocular responses to tilt and translation (Hageman et al., 2020), indicating successful stimulation of sections of the otolith sensitive to different axes of head motion. However, precisely positioning the arrays within the otoliths is difficult, requiring surgical approaches that risk damaging other structures. Furthermore, current spread across an implanted otolith limited both the magnitude and spatial selectivity of the device. Thus, significant work remains to be done in developing an otolith prosthesis.

### Safe direct current stimulation

Another research direction that could restore vestibular information to the brain in a more naturalistic way is the use of direct current (DC) stimulation, which has gained popularity in recent years for use in transcutaneous and transcranial stimulation (reviewed in Aplin and Fridman, 2019; Fridman and Della Santina, 2013). Unlike pulsatile stimulation that can only evoke more firing in the targeted nerve (and thus requires baseline stimulation rate in order to encode motion in opposing directions), DC stimulation can both increase and decrease the firing. However, DC stimulation is not generally used due to its electrochemical reactions that are harmful to tissue when used with traditional metal electrodes. Rather than directly stimulating tissue with a metal electrode, safe DC stimulators make use of ionic currents in saline with a switching mechanism that allows for continuous DC flow while also maintaining charge balance at the tissue interface (Figure 11B). In the context of the vestibular prosthesis, safe DC stimulation of the semicircular canals has been shown to evoke robust VOR eye movements consistent with the canal plane being stimulated in chinchillas (Aplin et al., 2019). Specifically, cathodic and anodic currents evoked eye movement in the excitatory and inhibitory (opposite) directions, respectively. Importantly, when compared to traditional pulsatile stimulation, DC stimulation provided a larger dynamic range of the evoked eye movements due to the ability to also inhibit activity. Additionally, electrophysiology (Manca et al., 2019) and modeling (Steinhardt and Fridman, 2021) studies have both shown that DC stimulator modulates afferent firing rate in a manner that also maintains their firing properties (i.e. irregularity and asynchronous behavior of firing). Critically, this preservation of firing properties would result in a more naturalistic activation of the afferent population, which could reduce any adverse effects resulting from sustained pulsatile stimulation (e.g. Figure 9).

Because DC stimulation does not modulate afferent activity in a manner that directly links stimulation pulses to the afferent firing rate (like pulsatile stimulation does), computing the current modulation that yields the desired naturalistic encoding requires knowledge of the relationship between stimulation and neuronal output. Fortunately, recent studies, quantifying afferent responses to transcranial DC stimulation of the vestibular system (i.e. galvanic vestibular stimulation), have shown that a transfer function between current delivered and afferent firing can be readily constructed and accounted for to evoke desired firing and head motion encoding within the afferents (Forbes et al., 2020; Forbes et al., 2023; Kwan et al., 2019; Figure 11C and D). Critically, this finding underscores how neurophysiological insights can directly guide translational and clinical applications. Ultimately, safe DC stimulation may become a stimulator of choice for vestibular prosthesis (alone or together with pulsatile stimulation) as it provides a more naturalistic way to control neural firing. Currently, the size and complexity of the required hardware limit the application of DC stimulation in the clinic, nevertheless, this remains an exciting area of active research.

### Conclusion

The unique properties of the vestibular system have driven rapid progress in the development of vestibular prostheses in recent years, both in animal models and clinical trials. These devices have shown promising functional outcomes, significantly improving the quality of life for patients who previously could not compensate through rehabilitation exercises alone. Such patients would otherwise continue to experience unsteadiness while walking or standing, blurred vision (oscillopsia) during head movements, or worsening instability in low-light conditions or uneven terrain. However, to further advance this technology, it is essential to fully incorporate our current understanding of vestibular neurophysiology into both prosthesis design and clinical application. A key step forward lies in leveraging natural afferent encoding strategies to tailor treatments to each patient’s specific needs. Personalized prosthesis mappings could be developed to target the most debilitating symptoms. For instance, patients primarily concerned with visual stability could benefit from a regular afferent mapping, while those focused on improving postural stability might see better results with an irregular afferent mapping (Figure 5). Nevertheless, it is also important to recognize that since current pulsatile prostheses will coincidentally stimulate all afferent types, addressing one problem with a specific mapping could potentially impair other functions that rely on a different mapping. While the full potential of this personalized approach has yet to be explored, the selection of afferent encoding during a patient’s initial ‘device activation’ visit may become a key aspect of prosthesis customization. Additionally, we note that future advancements in surgical techniques, though outside the scope of this review, will play a pivotal role in further enhancing vestibular prosthesis outcomes, as highlighted in a recent review by Stultiens et al., 2023.

Aside from furthering and leveraging our understanding of vestibular neurophysiology, gaining even more insights into how neural populations, along each stage of vestibular processing, respond to prosthetic stimulation will be key for addressing current limitations of prosthesis designs and clinical applications. In particular, future efforts should explore how higher-order brain areas, including the cerebellum, ascending thalamocortical pathways (i.e. the anterior head direction network and posterior thalamic pathway), and cortex respond and adapt to prosthetic inputs. Once the innovative engineering and the foundational neurophysiology are fully integrated, we believe the field will move forward and ultimately improve patient outcomes.
